# The potential impact of increased treatment rates for alcohol dependence in the United Kingdom in 2004

**DOI:** 10.1186/1472-6963-14-53

**Published:** 2014-02-05

**Authors:** Kevin D Shield, Jürgen Rehm, Maximilien X Rehm, Gerrit Gmel, Colin Drummond

**Affiliations:** 1 Centre for Addiction and Mental Health (CAMH), 33 Russell Street, Toronto, ON M5S 2S1, Canada; 2 Institute of Medical Science, University of Toronto, Toronto, Canada; 3 Institute for Clinical Psychology and Psychotherapy, TU Dresden, Germany; 4 Dalla Lana School of Public Health (DLSPH), University of Toronto, Toronto, Canada; 5 Department of Psychiatry, University of Toronto, Toronto, Canada; 6 Faculty of Arts and Sciences/Politics and Governance, Ryerson University, Toronto, Toronto, Canada; 7 National Addiction Centre, Institute of Psychiatry, King’s College London, London, UK

**Keywords:** Alcohol, Mortality, Alcohol dependence, Alcohol dependence treatment, United Kingdom

## Abstract

**Background:**

Alcohol consumption has been linked to a considerable burden of disease in the United Kingdom (UK), with most of this burden due to heavy drinking and Alcohol Dependence (AD). However, AD is undertreated in the UK, with only 8% of those individuals with AD being treated in England and only 6% of those individuals with AD being treated in Scotland. Thus, the objective of this paper is to quantify the deaths that would have been avoided in the UK in 2004 if the treatment rate for AD had been increased.

**Methods:**

Data on the prevalence of AD, alcohol consumption, and mortality were obtained from the Adult Psychiatric Morbidity Survey, the Global Information System on Alcohol and Health, and the 2004 Global Burden of Disease study respectively. Data on the effectiveness of pharmacological treatment and Motivational Interviewing/Cognitive Behavioural Therapy were obtained from Cochrane reviews and meta-analyses. Simulations were used to model the number of deaths under different treatment scenarios. Sensitivity analyses were performed to model the effects of Brief Interventions and to examine the effect of using AD prevalence data obtained from the National Institute for Health and Clinical Excellence.

**Results:**

In the UK, 320 female and 1,385 male deaths would have been avoided if treatment coverage of pharmacological treatment had been increased to 20%. This decrease in the number of deaths represents 7.9% of all alcohol-attributable deaths (7.0% of all alcohol-attributable deaths for women and 8.1% of all alcohol-attributable deaths for men). If we used lower AD prevalence rates obtained from the National Institute for Health and Clinical Excellence, then treatment coverage of pharmacological treatment in hospitals for 20% of the population with AD would have resulted in the avoidance of 529 deaths in 2004 (99 deaths avoided for women and 430 deaths avoided for men).

**Conclusions:**

Increasing AD treatment in the UK would have led to a large number of deaths being avoided in 2004. Increased AD treatment rates not only impact mortality but also impact upon the large burden of disability and morbidity attributable to AD, as well as the associated social and economic burdens.

## Background

Alcohol consumption causes a large burden of disease [1,2], with most of this burden caused by heavy drinking and Alcohol Dependence (AD) [3], where heavy drinking is defined as consuming a daily average of 5 or more standard United Kingdom (UK) drinks for women (40+ grams (g) of pure alcohol) and consuming a daily average of 7.5 standard UK drinks or more for men (60+ g of pure alcohol) [4]. In the European Union (EU) in 2004 for people 15 to 64 years of age, it has been estimated that 77.3% of the net alcohol-attributable burden of mortality, 67.2% of the detrimental alcohol-attributable burden (i.e., the burden not including the beneficial effects of alcohol for selected ischemic disease categories and diabetes), and 9.2% of the total burden of mortality resulted from heavy drinking [3]. One of the most severe consequences of alcohol consumption is AD (defined as a maladaptive behaviour of drinking of alcoholic beverages with clinically relevant consequences) [5]. AD is strongly associated with frequency of heavy drinking episodes [6,7].

### Alcohol consumption in the UK

In the UK in 2009 the average adult *per capita* consumption was 12.5 litres (l), the same as the EU (12.5 l), 89.3% of women and 91.3% of men were current drinkers (people who consumed alcohol in the past year) [8], and 8.9% of women and 15.5% of men were heavy drinkers [8]. Patterns of drinking in the UK are quite detrimental, as indicated by the UK’s pattern of drinking score of 3 (out of 5) in 2009, with a high prevalence of regular and irregular heavy drinking occasions [8]. In the UK in 2009 approximately 14% of drinkers consumed 5 or more drinks several times a week, 20% consumed 5 or more drinks once a week, and a further 14% consumed 5 or more drinks once a month [9].

### Alcohol dependence in the UK

In England in 2007 approximately 3.6% of women and 9.3% of men 15 to 64 years of age had AD [10]; this translates into more than 2 million people afflicted with AD in England. The National Institute for Health and Clinical Excellence estimated the prevalence of AD to be 2% for women and 6% for men [11]. By comparison, the prevalence of AD in 2005 in the EU was lower than in the UK, with 1.5% and 5.4% for women and men 15 to 64 years of age respectively being alcohol dependent [8].

### Alcohol-attributable mortality in the UK

In the UK in 2004 an estimated 13.1 female and 27.6 male deaths (per 100,000 people) among people 15 to 64 years of age were attributable to alcohol consumption [12], with 6.4% 8.5% of all premature deaths among women and men respectively being caused by alcohol consumption. In other words, 1 out of 16 and 1 out of 12 premature deaths of women and men respectively were caused by alcohol consumption.

### Alcohol dependence treatment in the UK

Unfortunately, while there are effective treatment options available, both in terms of psychosocial [13] and pharmacological interventions for AD [14,15], the overall treatment rate for AD is low (for an overview of various treatment interventions for AD, see [11]). In Europe, less than 10% of all people with AD receive treatment in any given year [8,16], and in England it is estimated that 6% of the population 15 to 64 years of age with AD receive treatment in any given year [17]. In Scotland, where prevalence of AD is higher than in England, treatment coverage is over 8% [18]. Therefore, given the burden of AD in the UK and the low treatment coverage rates, increasing treatment rates and applying evidence-based psychosocial and pharmacological interventions could result in considerable reductions in alcohol-attributable mortality in the UK [3]. Thus, the aim of this article is to quantify the effect of increasing AD treatment coverage rates in the UK.

## Methods

### Data sources

Data on drinking status for the UK were obtained from the Global Status Report on Alcohol and Health [19]; for the UK the drinking status estimates were based on large population surveys and government statistics. Data on binge drinking patterns were obtained from the European Commission report and from the World Health Organization [9,19]. *Per capita* consumption of alcohol data were obtained from the Global Information System on Alcohol and Health ( http://www.who.int/gho/alcohol/en/index.html). Pattern of drinking score data were obtained from the 2010 Global Burden of Disease (GBD) study (see [20] for data on drinking pattern scores and *per capita* consumption by country for 2005). The pattern of drinking score, developed as part of the Comparative Risk Assessment for alcohol within the GBD studies, is a composite measure based on frequency of heavy drinking occasions, the amount consumed per occasion, the proportion of overall consumption due to drinking to intoxication, and the proportion of drinking occasions in combination with meals (see [21] for a definition of patterns of drinking and the construction of a comparative score). Data on AD were obtained from [10], and additional estimates of AD for the sensitivity analyses were obtained from the National Institute for Health and Clinical Excellence [11]. Mortality data by cause, age, and sex for the UK for 2004 were obtained from the 2004 GBD study [4].

### Modelling alcohol consumption

The drinking prevalence of any population can be estimated using sex- and age-specific *per capita* consumption data; this data is used to derive a Gamma distribution which is used to model the distribution of alcohol consumption. This continuous prevalence distribution, combined with continuous Relative Risk (RR) functions, is used to derive the proportion of deaths attributable to alcohol consumption (the proportion of deaths that would not be present under a counterfactual scenario where no one consumed alcohol) [22,23].

### Modelling the effect of interventions for AD on mortality

Given the low current rate of treatment for people with AD in the UK, we simulated the potential effects of the following interventions: 1) pharmacological treatment 2) Motivational Interviewing/Cognitive Behavioural Therapy (MI/CBT) and 3) Brief Interventions (BI) (the effects of BI were only used for sensitivity analyses as the estimated effects of BI have limitations (see limitations section) (for a detailed description of the methodology, see Additional file 1).

The effect of treatment interventions for AD can be expressed as a reduction in average alcohol consumption or a decrease in the risk of mortality. To estimate the effect of increasing treatment coverage for AD on mortality, we applied a reduction in alcohol consumption to a subset of the population with AD, and compared the estimated Alcohol-Attributable Fractions (defined as the proportion of mortality that would not have occurred if people had never consumed alcohol [24]) for the entire population before and after the increase in AD treatment coverage rates.

To estimate the effect of MI/CBT, an average drop in consumption of 15.8 g of pure alcohol per day was assumed (measured against no intervention (using the 95% Confidence Interval (CI) of 9.6 g to 21.8 g)) [25,26]. As an upper limit of the effect of MI/CBT, we modelled the effect using an average drop in consumption of 21.8 g of pure alcohol per day, i.e., the upper limit of the 95% CI. We combined CBT and MI, as the meta-analyses on their effectiveness yielded almost identical results. In addition, Project Matching alcoholism treatment to client heterogeneity did not observe any significant differences between the use of either MI or CBT [27]. To estimate the effects of pharmacological therapy for AD, we combined the effects of randomized controlled trials for acamprosate and opioid antagonist therapy [14,15] by calculating the difference in alcohol consumption between baseline and follow-up in the group that received medication. The estimated effect was for the patient population that received the pharmacological therapy; 55.0% reduced their alcohol consumption by an average of 13% (18.1% of the patient population reduced their drinking by 50% and 26.8% of the population achieved abstinence).

To estimate the effect of BI in hospital settings, two different estimates were modelled. The first approach was based on an estimated reduction in consumption of 13.5 g of pure alcohol per day from the meta-analysis (using a 95% CI of 2.7 g to 24.5 g) [28,29] (this treatment is represented by the term BI (1)). The second approach was to assume an average RR for mortality of 0.6 (95% CI of 0.40 to 0.91) for people with AD who received BI therapy [28] (this treatment is represented by the term BI (2)). This RR for mortality for people who are alcohol dependent was obtained from the Cochrane review of all studies which had a 12 month follow-up [28].

The drinking population was modelled using 100,000 samples drawn from the Gamma distribution representing the drinking population of the UK. It was assumed that only heavier drinkers would receive interventions, as their identification as being alcohol dependent is more likely. The people with AD were, therefore, randomly selected among the samples displaying an average alcohol consumption of 72 g and 48 g of pure alcohol per day or more for men and women respectively. This lower limit was based on alcohol consumption among people with AD [30].

The number of deaths avoided under different treatment scenarios was calculated by estimating the number of alcohol-attributable deaths at the population level, assuming 0% of people with AD were receiving treatment, and then by comparing this estimate to a simulated population where 10%, 20%, 30% and 40% of people with AD were receiving AD treatment. Alcohol-attributable mortality was calculated by applying alcohol dose and disease specific relative risks for mortality to each of the 100,000 random samples (see Additional file 2 for the methodology used to estimate the mortality attributable to AD).

The main analyses and sensitivity analyses using alternative AD prevalence data were performed using the above-described methods. The sensitivity analyses differ only with respect to the AD prevalence data that were used.

No ethics approvals were required, as our analyses were considered secondary data of existing databases.

## Results

### Impact of increasing treatment rates for AD on mortality

**Figure 1:**
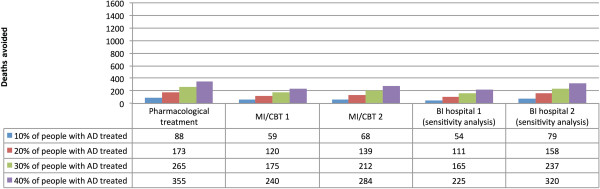
Deaths avoided in the UK for women based on different treatment coverage rates for AD for pharmacological treatment, Motivational Interviewing/Cognitive Behavioural (MI/CBT) (based on the lower (MBI/CT 1) and upper reported estimates (MBI/CT 2)), and Brief Interventions (BI) treatment (based on the resulting reduction in alcohol consumption (BI hospital 1) and the resulting reduction in mortality (BI hospital 2)).

**Figure 2:**
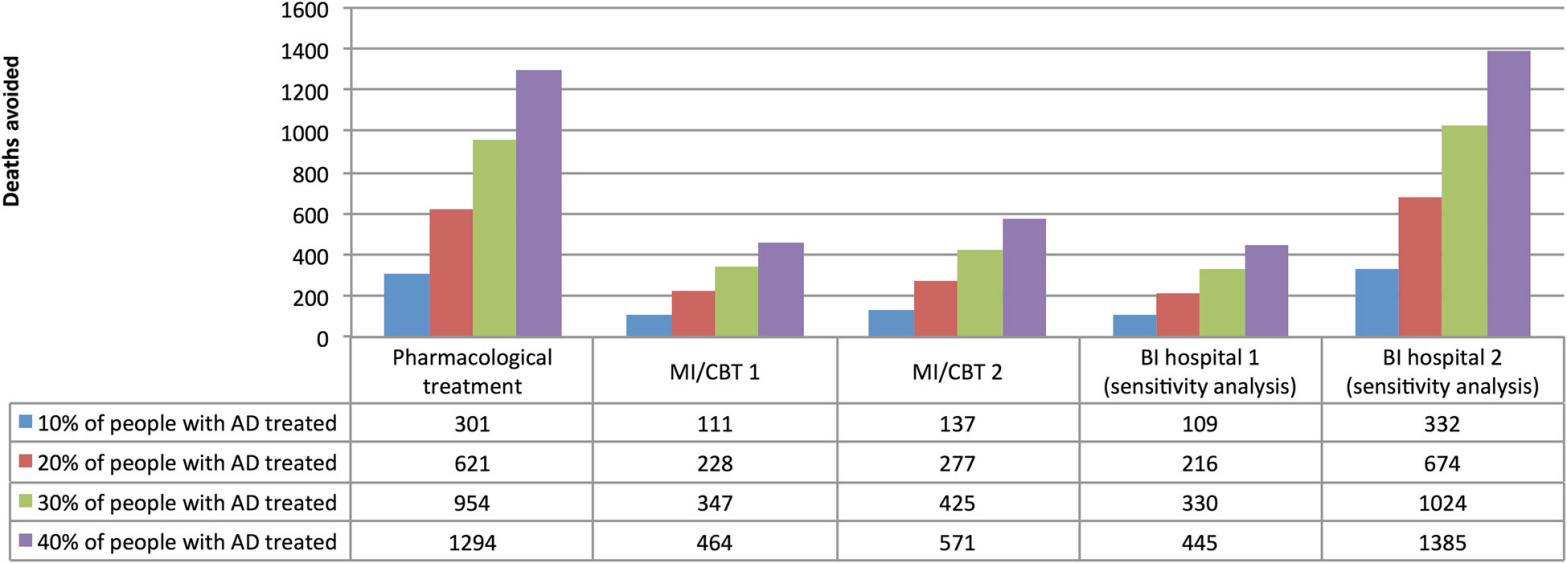
Deaths avoided in the UK for men based on different treatment coverage rates for AD for pharmacological treatment, Motivational Interviewing/Cognitive Behavioural (MI/CBT) (based on the lower (MBI/CT 1) and upper reported estimates (MBI/CT 2)), and Brief Interventions (BI) treatment (based on the resulting reduction in alcohol consumption (BI hospital 1) and the resulting reduction in mortality (BI hospital 2)).

Pharmacological therapy was the most effective intervention modelled at a AD treatment coverage rate of 20%, with an estimated 794 deaths avoided in 2004 (173 deaths avoided among women and 621 deaths avoided among men), representing 7.9% of all alcohol-attributable deaths (7.0% of all alcohol-attributable deaths for women and 8.1% of all alcohol-attributable deaths for men). The second and third most effective interventions at a coverage rate of 20% were MI/CBT (higher effectiveness), with an estimated 416 deaths avoided, and MI/CBT, with an estimated 348 deaths avoided respectively. The numbers of deaths avoided under the conditions that 10%, 20%, 30% and 40% of people with AD were treated are outlined in Figure 1 for women and Figure 2 for men by AD treatment type. See Additional file 3 for the percentage of alcohol-attributable deaths prevented using the alternative AD prevalence rates.

### Sensitivity analyses

If BI (modelled using a reduction in the risk of mortality, namely BI (2)) were implemented for 20% of the population, 832 deaths would be avoided in 2004 (158 deaths avoided for women and 674 deaths avoided for men), representing 8.2% of all alcohol-attributable deaths (6.5% of all alcohol-attributable deaths for women and 8.8% of all alcohol-attributable deaths for men). The effects of BI were greater than those of any other intervention.

**Figure 3:**
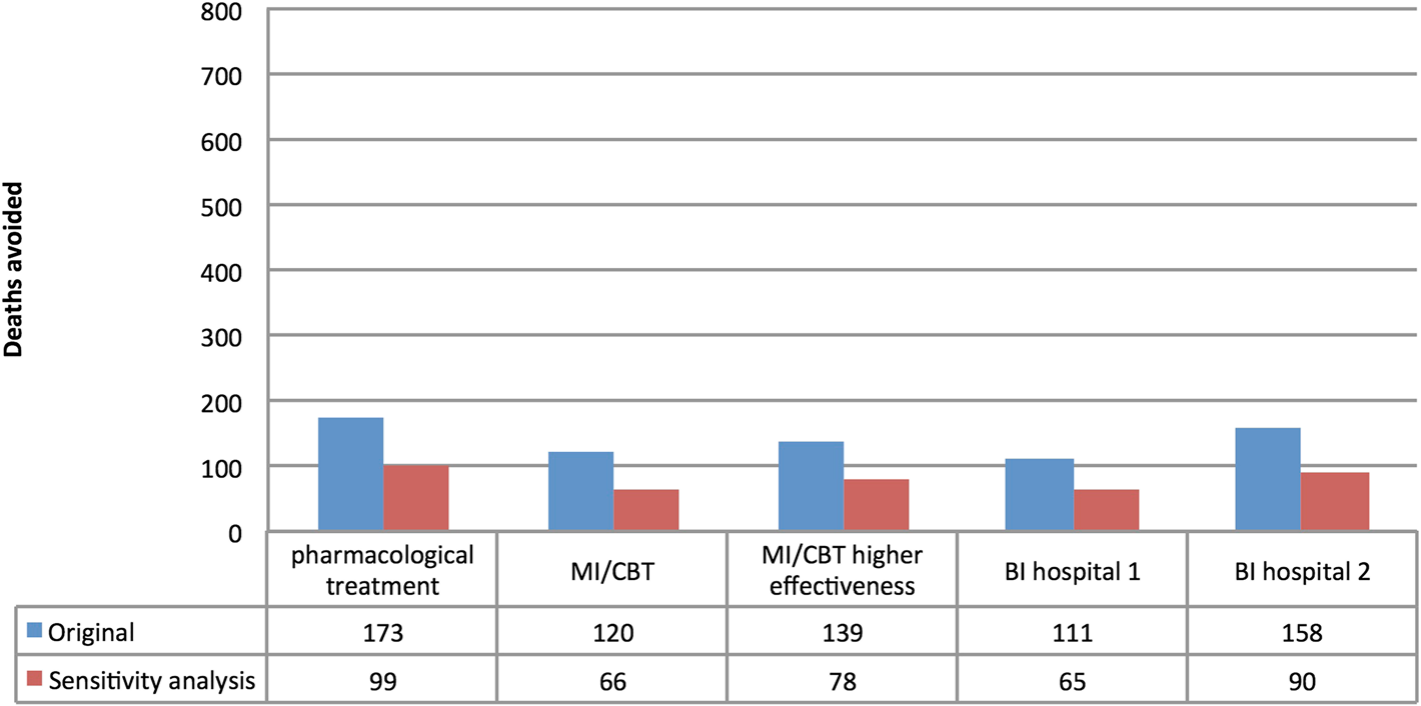
Number of deaths avoided in the UK for women based on two different prevalence estimates for AD (assuming a 40% treatment coverage rate) for pharmacological treatment, Motivational Interviewing/Cognitive Behavioural (MI/CBT) (based on the lower (MBI/CT 1) and upper reported estimates (MBI/CT 2)), and Brief Interventions (BI) treatment (based on the resulting reduction in alcohol consumption (BI hospital 1) and the resulting reduction in mortality (BI hospital 2)).

**Figure 4:**
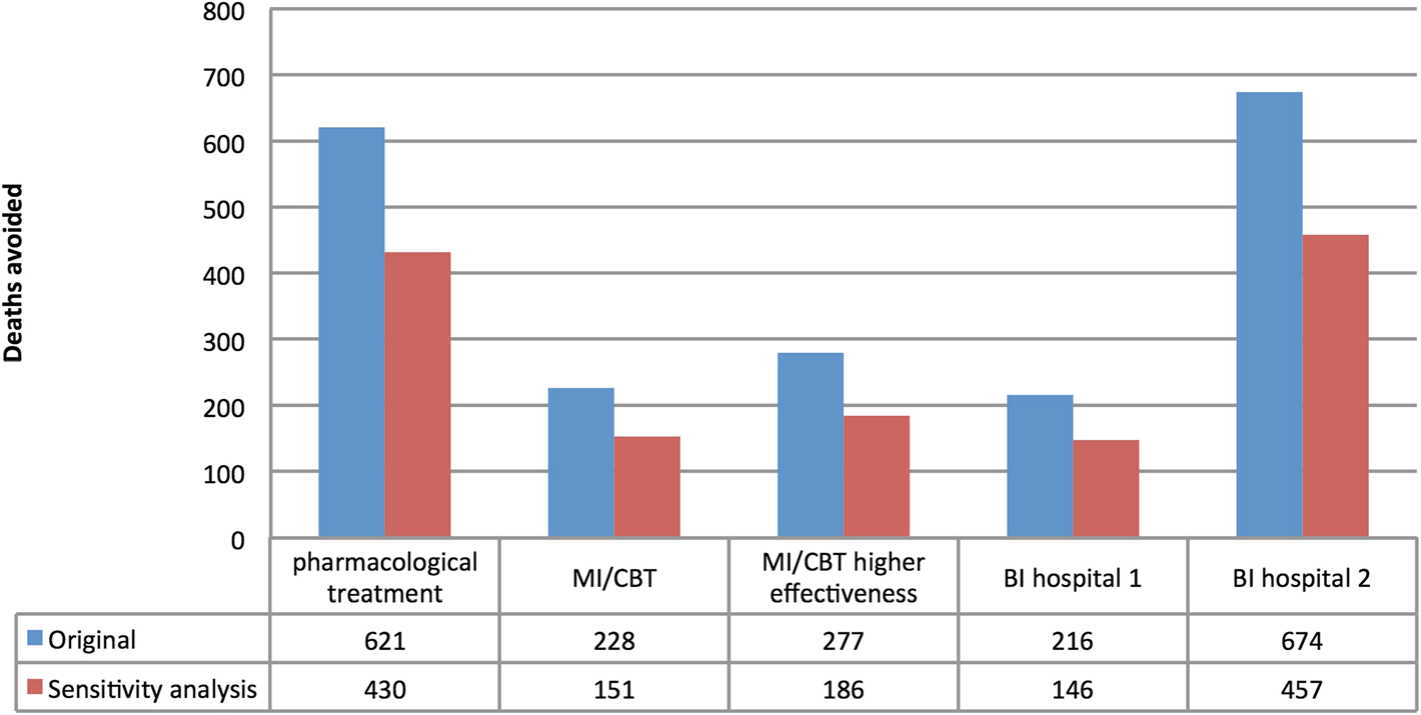
Number of deaths avoided in the UK for men based on two different prevalence estimates for AD (assuming a 40% treatment coverage rate) for pharmacological treatment, Motivational Interviewing/Cognitive Behavioural (MI/CBT) (based on the lower (MBI/CT 1) and upper reported estimates (MBI/CT 2)), and Brief Interventions (BI) treatment (based on the resulting reduction in alcohol consumption (BI hospital 1) and the resulting reduction in mortality (BI hospital 2)).

When the lower prevalences of AD and treatment coverage were modelled under the scenario where 20% of people with AD would be treated, pharmacological treatment was the most effective main intervention modelled (529 deaths avoided; 99 deaths avoided for women, and 430 deaths avoided for men), followed by MI/CBT (assuming a higher level of effectiveness) (264 deaths avoided), and then MI/CBT (217 deaths avoided). In the sensitivity analysis, BI (2) was the more effective than any of the main interventions modelled (547 deaths avoided). A summary of the comparison between the main and the alternative scenarios can be found in Figures 3 and 4.

## Discussion

AD is an important contributor to the burden of disease in the UK, and a large proportion of this burden could be reduced if the treatment coverage rate for AD was increased. Additionally, treatment of AD is important in the UK, as the burden of alcohol-attributable mortality is high [12] and has increased over the past 20 years in key categories such as liver disease [31]. Interestingly, even though overall average *per capita* consumption is the same in the UK as in the rest of the EU, the proportion of the burden of disease attributable to alcohol consumption is higher for men and even more so for women in the UK when compared to the rest of the EU [3,12]. This apparent contradiction may be due to the fact that people consume alcohol in the UK in a more detrimental manner than their EU counterparts [32].

An increase in AD treatment rates will not only impact mortality, but also will impact upon the large burden of disability and morbidity attributable to AD, as well as the associated social and economic burdens [33-36]; however, increasing the treatment coverage for AD is non-trivial. One way to increase treatment coverage for AD is by increasing treatment options, such as offering the reduction of alcohol consumption as a treatment outcome [37]. A second way of increasing treatment coverage for AD is to reduce the stigma associated with AD [37]. The third way to increase treatment coverage is to ensure identification of individuals with AD when they come into contact with the medical system, such as during family physician (general practitioner) visits, and then be treated by a general practitioner for less severe cases of AD or referred to AD treatment services for more severe cases of AD [38]. All of these methods are difficult to implement and potentially costly [38,39]. Additionally, increasing treatment coverage of AD is not cost-effective when compared to other interventions, such as increasing taxation [29,38,40]. However, given the need to increase health equity as mandated by the World Health Organization, and the observation that people with AD are more likely to be members of a racial minority group and/or to have a low-income, increasing AD treatment coverage rates is required from a health equity perspective [41].

### Limitations

There are limitations to the methodology for the estimated number of deaths avoided if the treatment coverage for AD was increased to 40%. First, the results for BI were based on male heavy drinkers (with some studies included in the meta-analysis excluding people with AD) and, thus, the applicability of these results to men and especially to women with AD is questionable [28]. However, it should be noted that the association between heavy drinking and AD is strong [42], and heavy drinking has been suggested as main domain for the operationalization for alcohol use disorders [43].

Second, the high estimates of the effects of the BIs are based on the latest Cochrane analyses of seven randomized controlled clinical trials (RCTs) in hospitals [28]. It is plausible that a relatively short intervention can have a large effect in this population, as people in hospitals have higher risks of premature mortality, and the reduction of alcohol consumption in such a group has shown important effects on mortality (for an overview see [8]). Thus, BI in hospitals represents a "best case" scenario, as AD treatment plays an important role in mediating and moderating premature mortality (e.g. [44,45]). However, effects on mortality were also observed in a meta-analyses of BIs in all settings and populations [46]. Therefore, we used the results from the meta-analysis by McQueen and colleagues on BIs as a sensitivity analysis only, as these results may not represent the actual number of deaths avoided if treatment coverage for BIs was increased [28].

The third limitation to our study is that even though overall average *per capita* consumption is the same in the UK as in the rest of the EU, the proportion of the burden of disease attributable to alcohol consumption is higher for men and even more so for women in the UK when compared to the rest of the EU [3,11]. The reason for this contradiction may be due to the fact that people consume alcohol in the UK in a more detrimental manner than their EU counterparts [34]. Additionally, this study modelled only the effect of increasing the treatment coverage rate for AD on mortality. Increasing AD treatment rates will also impact the large burden of disability and morbidity attributable to AD, as well as the associated social and economic burdens [35-38]. Fourth, while we estimated the effects for one year, most of the RCTs had smaller follow-up times, and the effects may not be as strong for one year. This may especially affect the psychotherapies, but does not affect the estimated reduction in the RR of mortality from BI in hospitals as this reduction was based only on RCTs with a follow-up of one year. This limitation may be as a result of differences between efficacy and effectiveness [47-49] of trials, i.e., not all of the effects seen in RCTs in selected populations may transfer into the real world (see also [50]). Additionally, it is unclear if BI would be as effective if the coverage of alcohol interventions was increased to 40% of the population with AD, a proportion of whom may be less motivated to engage in the interventions offered compared to those currently receiving treatment.

A fifth limitation is the feasibility of increasing the treatment rate to 40%, as a similar coverage rate has not been achieved in any other similar high-income country [3]. The final limitation to our study is that the analysis was limited to single AD interventions, and the effects of combination therapies have not been examined through a meta-analysis [3].

## Conclusions

The burden of mortality attributable to alcohol consumption in the UK is large, and has been increasing over the past 20 years in key categories such as liver cirrhosis. A large proportion of the burden of disease in the UK is attributable to unhealthy patterns of alcohol consumption and to AD. Based on the analysis of data for 2004, this article presents observations that an increase in AD treatment in the UK could lead to the prevention of a large number of deaths.

## Abbreviations

AD: Alcohol dependence; BI: Brief interventions; CI: Confidence interval; EU: European Union; GBD: Global burden of disease; g: grams; l: litre(s); MI/CBT: Motivational interviewing/cognitive behavioural therapy; RCT: Randomized controlled clinical trials; RR: Relative risk; UK: United Kingdom.

## Competing interests

Lundbeck provided financial support for this study via an unrestricted grant. Lundbeck had no influence on the data gathering or design of this study. In the past few years Jürgen Rehm has received unrestricted grants from Eli Lilly, Schering-Plough, and Lundbeck, and is a member of the scientific advisory council for Nalmefene.

## Authors’ contributions

JR and CD conceptualized the overall article. KDS, JR, MXR, and GG acquired all data. All authors contributed to the methodology. KS and GG performed all statistical analyses. All authors contributed to the writing of the manuscript and approved the final version.

## Pre-publication history

The pre-publication history for this paper can be accessed here:

http://www.biomedcentral.com/1472-6963/14/53/prepub

## Supplementary Material

Additional file 1 Modelling the effects of AD interventions. Click here for file

Additional file 2 Modelling mortality attributable to alcohol consumption. Click here for file

Additional file 3 Percentage of all alcohol-attributable deaths avoided by increasing AD treatment coverage to 20% (sensitivity analyses). Click here for file
