# Supplementary material for: The potential impact of increased treatment rates for alcohol dependence in the United Kingdom in 2004
Source: BMC Health Serv Res. 2014 Feb 5;14:53. doi: 10.1186/1472-6963-14-53 (PMC3923387; doi:10.1186/1472-6963-14-53)
Supplement: Additional file 1 — Modelling the effects of AD interventions. [file 1472-6963-14-53-S1.docx]

## Additional file 1. Modelling the effects of AD interventions

| **Interventions** | **Main results (effects assumed to be stable for 1 year)** | **Risk Relations** | **Sources** |
| --- | --- | --- | --- |
| **MI and CBT 1** | To model the effect of MI we assumed an average drop of 15.8 g of pure alcohol per day was assumed (measured against no intervention; 95% (Confidence Interval (CI) from -9.6 g to -21.8 g of pure alcohol). The effect after one year was very small and not significant (average: 1.2 g of pure alcohol reduction per day), the average effect over the year was a 3.2 g reduction of pure alcohol per day (95% CI: -1.2 g to -5.2 g of pure alcohol per day).  For CBT almost the same effect was found in studies with a no-treatment control as the comparison condition (15.9 g of pure alcohol per day). In addition, Project Match did not find any significant differences [[1](#_ENREF_1)] between MI and CBT. Thus we modelled the results based on a drop of 15.8 g per day over the year. | The usual dose-dependent risk relations between average consumption of alcohol and disease outcomes were used, multiplied by 2 to account for the overall higher mortality risk of people with AD according to the findings of Harris and Barraclough [[2](#_ENREF_2)]. For injury, the RR from [[2](#_ENREF_2)] was used for AD and the risks from [[3](#_ENREF_3)] for non-dependent people. | MI: [[4](#_ENREF_4)]  CBT: [[5](#_ENREF_5)] |
| **MI and CBT 2** | An average drop of 21.8 g of pure alcohol per day was assumed as the upper limit of the CI for MI/CBT (see above). We assumed proportional CIs compared to the first MI/CBT scenario. |  | [[4](#_ENREF_4)] |
| **BI 1 (Sensitivity analysis only)** | To model the effect of BI an average drop of 13.5 g of pure alcohol per day with a 95% CI from -2.7 to -24.5 of pure alcohol per day. |  | [[6](#_ENREF_6), [7](#_ENREF_7)] |
| **BI 2 (Sensitivity analysis only)** | An average reduction of the RR for mortality by 0.6 (95% CI: 0.40 to 0.91). This scenario represents the “best case” for BI, as hospitalization is linked to mortality, and AD plays an important role in mediating and moderating this premature mortality (e.g. [[8](#_ENREF_8), [9](#_ENREF_9)]). However, similar effects were obtained in an meta-analyses on all BIs [[10](#_ENREF_10)]. |  | [[6](#_ENREF_6)] |
| **Pharmacological therapy (for simulation, the effects of Randomized Controlled Trials of acamprosate and opioid antagonist treatments were combined** | To model the effect of pharmacological therapy Overall, for 55.0% of the patient population a reduction in drinking by 13% on average; for 18.1% of the patient population there was a reduction in drinking by 50%; and for 26.8% of the population abstinence was the result. |  | Pooled estimates of [[11](#_ENREF_11), [12](#_ENREF_12)]. For this simulation we are concerned with the differences in consumption between baseline and follow-up in the group receiving medications only. |

Reference List

1. Project Match Research Group: **Matching alcoholism treatment to client heterogeneity: Project MATCH posttreatment drinking outcomes**. *J Stud Alcohol* 1997, **58**:7-30.

2. Harris EC, Barraclough B: **Excess mortality of mental disorder**. *Br J Psychiatry* 1998, **173**:11-53.

3. Corrao G, Bagnardi V, Zambon A, La Vecchia C: **A meta-analysis of alcohol consumption and the risk of 15 diseases**. *Prev Med* 2004, **38**:613-619.

4. Smedslund G, Berg RC, Hammerstrom KT, Steiro A, Leiknes KA, Dahl HM, Karlsen K: **Motivational interviewing for substance abuse**. *Cochrane Database Syst Rev* 2011, **5**:CD008063.

5. Magill M, Ray LA: **Cognitive-behavioral treatment with adult alcohol and illicit drug users: a meta-analysis of randomized controlled trials**. *J Stud Alcohol Drugs* 2009, **70**(4):516-527.

6. McQueen J, Howe TE, Allan L, Mains D, Hardy V: **Brief interventions for heavy alcohol users admitted to general hospital wards**. *Cochrane Database Syst Rev* 2011, **8**:CD005191.

7. Room R, Babor T, Rehm J: **Alcohol and public health: a review**. *Lancet* 2005, **365**:519-530.

8. O'Brien JM, Jr., Lu B, Ali NA, Martin GS, Aberegg SK, Marsh CB, Lemeshow S, Douglas IS: **Alcohol dependence is independently associated with sepsis, septic shock, and hospital mortality among adult intensive care unit patients**. *Crit Care Med* 2007, **35**(2):345-350.

9. De Lorenze GN, Weisner C, Tsai AL, Satre DD, Quesenberry CP, Jr.: **Excess mortality among HIV-infected patients diagnosed with substance use dependence or abuse receiving care in a fully integrated medical care program.** *Alcohol Clin Exp Res* 2011, **35**(2):203-210.

10. Cuijpers P, Riper H, Lemmers L: **The effects on mortality of brief interventions for problem drinking: a meta-analysis**. *Addiction* 2004, **99**(7):839-845.

11. Rösner S, Hackl-Herrwerth A, Leucht S, Lehert P, Vecchi S, Soyka M: **Acamprosate for alcohol dependence**. *Cochrane Database Syst Rev* 2010, **9**:CD004332.

12. Rösner S, Hackl-Herrwerth A, Leucht S, Vecchi S, Srisurapanont M, Soyka M: **Opioid antagonists for alcohol dependence**. *Cochrane Database Syst Rev* 2010, **12**:CD001867.
