# Supplementary material for: The potential impact of increased treatment rates for alcohol dependence in the United Kingdom in 2004
Source: BMC Health Serv Res. 2014 Feb 5;14:53. doi: 10.1186/1472-6963-14-53 (PMC3923387; doi:10.1186/1472-6963-14-53)
Supplement: Additional file 2 — Modelling mortality attributable to alcohol consumption. [file 1472-6963-14-53-S2.docx]

**Additional file 2. Modelling mortality attributable to alcohol consumption**

The number of alcohol-attributable deaths was calculated by sex according to the following formula [[1](#_ENREF_1)]:

$$Mortality=\sum_{a}^{n\_a} \left[Mortality_{cause\_a}*\left(\frac{\sum_{b}^{N\_b} RR_{Norm\_a}(x)_{Norm\_b}+\sum_{c}^{N\_c} RR_{AD\_a}(x)_{AD\_c}-1}{\sum_{b}^{N\_b} RR_{Norm\_a}(x)_{Norm\_b}+\sum_{c}^{N\_c} RR_{AD\_a}(x)_{AD\_c}}\right)\right]$$

where “a” represents an alcohol related cause of death and “n_a” represents the total number of alcohol related diseases for which the alcohol-attributable mortality can be calculated (see table A1), and “Mortality_cause_a_” represents the mortality for cause “a”. “N_b” represents the number of people who do not have Alcohol dependence (AD) (“b”) and “N_c” represents the number of people who do have AD (“c”). For the Relative Risks (RR), “RR_Norm_a_(x)_Norm_b_” represents the RR for the alcohol related disease or condition “a” given an average daily alcohol consumption of x for person “b”, and “RR_AD_a_(x)_Norm_c_” represents the RR for the alcohol related disease or condition “a” given an average daily alcohol consumption of x for person “c”. For people with alcohol dependence the relative risk function was calculated by multiplying the RR for people for the general population by 2 to account for the overall higher mortality risk of people with AD (this correction factor was based on the RR findings of Harris & Barraclough [[2](#_ENREF_2)] )) . The references for the sources of the RR functions are outlined in table a1.

Table A1. The source of Relative Risk functions for each alcohol related disease used when calculating the number of deaths attributable to alcohol consumption.

| **Condition** | | **ICD 10 Code** | **Source for RR function** |
| --- | --- | --- | --- |
| **Infectious and parasitic diseases** | |  |  |
|  | Tuberculosis | A15-A19 | [[3](#_ENREF_3)]; for causal relationship see: [[4](#_ENREF_4)] |
| **Human immunodeficiency virus/ Acquired immune deficiency syndrome** | | B20-B24 | [[5](#_ENREF_5)] |
| **Malignant neoplasm's** | |  |  |
|  | Mouth and oropharynx cancers | C00-C14 | [[6](#_ENREF_6), [7](#_ENREF_7)] (based on Relative Risks from [[8](#_ENREF_8)]) |
|  | Esophageal cancer | C15 | [[6](#_ENREF_6), [7](#_ENREF_7)] (based on Relative Risks from [[8](#_ENREF_8)]) |
|  | Liver cancer | C22 | [[6](#_ENREF_6), [7](#_ENREF_7)] (based on Relative Risks from [[8](#_ENREF_8)]) |
|  | Laryngeal cancer | C32 | [[6](#_ENREF_6), [7](#_ENREF_7)] (based on Relative Risks from [[8](#_ENREF_8)]) |
|  | Breast cancer | C50 | [[6](#_ENREF_6), [7](#_ENREF_7)] (based on Relative Risks from [[8](#_ENREF_8)]) |
|  | Colon cancer | C18 | [[6](#_ENREF_6), [7](#_ENREF_7)] (based on Relative Risks from [[8](#_ENREF_8)]) |
|  | Rectal cancer | C20 | [[6](#_ENREF_6), [7](#_ENREF_7)] (based on Relative Risks from [[8](#_ENREF_8)]) |
| **Diabetes** | |  |  |
|  | Diabetes mellitus | E10-E14 | [[9](#_ENREF_9)] |
| **Neuro-psychiatric conditions** | |  |  |
|  | Alcoholic psychoses (part of AUD) | F10.0, F10.3-F10.9 | 100% AAF per definition |
|  | Alcohol abuse (part of AUD) | F10.1 | 100% AAF per definition |
|  | Alcohol dependence (part of AUD) | F10.2 | 100% AAF per definition |
|  | Epilepsy | G40-G41 | [[10](#_ENREF_10)] |
| **Cardiovascular disease** | |  |  |
|  | Hypertensive disease | I10-I15 | [[11](#_ENREF_11)] |
|  | Ischemic heart disease | I20-I25 | [[12](#_ENREF_12)], for volume, [[13](#_ENREF_13)] for pattern |
|  | Cardiac arrhythmias | I47-I49 | [[14](#_ENREF_14)] |
|  | Ischemic stroke | I60-I62 | [[15](#_ENREF_15)] |
|  | Haemorrhagic and other non-ischemic stroke | I63-I66 | [[15](#_ENREF_15)] |
| **Digestive diseases** | |  |  |
|  | Cirrhosis of the liver | K70, K74 | [[16](#_ENREF_16)] |
|  | Acute and chronic pancreatitis | K85, K86.1 | [[17](#_ENREF_17)] |
| **Respiratory infections** | |  |  |
|  | Pneumonia | J10.0, J11.0, J12-J15, J18 | [[18](#_ENREF_18)] |
| **Unintentional injuries** | |  |  |
|  | Motor vehicle accidents | § | [[2](#_ENREF_2)] for people with Alcohol Dependence (AD) and [[8](#_ENREF_8)] for people without AD |
|  | Poisonings | X40-X49 | [[2](#_ENREF_2)] for people with AD and [[8](#_ENREF_8)] for people without AD |
|  | Falls | W00-W19 | [[2](#_ENREF_2)] for people with AD and [[8](#_ENREF_8)] for people without AD |
|  | Fires | X00-X09 | [[2](#_ENREF_2)] for people with AD and [[8](#_ENREF_8)] for people without AD |
|  | Drowning | W65-W74 | [[2](#_ENREF_2)] for people with AD and [[8](#_ENREF_8)] for people without AD |
|  | Other Unintentional injuries | †Rest of V-series and W20-W64, W 75-W99, X10-X39, X50-X59, Y40-Y86, Y88, and Y89 | [[2](#_ENREF_2)] for people with AD and [[8](#_ENREF_8)] for people without AD |
| **Intentional injuries** | |  | [[2](#_ENREF_2)] for people with AD and [[8](#_ENREF_8)] for people without AD |
|  | Self-inflicted injuries | X60-X84 and Y87.0 | [[2](#_ENREF_2)] for people with AD and [[8](#_ENREF_8)] for people without AD |
|  | Homicide | X85-Y09, Y87.1 | [[2](#_ENREF_2)] for people with AD and [[8](#_ENREF_8)] for people without AD |
|  | Other intentional injuries | Y35 | [[2](#_ENREF_2)] for people with AD and [[8](#_ENREF_8)] for people without AD |
| § V021–V029, V031–V039, V041–V049, V092, V093, V123–V129, V133–V139, V143–V149, V194–V196, V203–V209, V213–V219, V223–V229, V233–V239, V243–V249, V253–V259, V263–V269, V273– V279, V283–V289, V294–V299, V304–V309, V314–V319, V324–V329, V334–V339, V344–V349, V354–V359, V364–V369, V374–V379, V384–V389, V394–V399, V404–V409, V414–V419, V424–V429, V434–V439, V444–V449, V454–V459, V464– V469, V474–V479, V484–V489, V494–V499, V504–V509, V514–V519, V524–V529, V534–V539, V544–V549, V554–V559, V564–V569, V574–V579, V584–V589, V594–V599, V604–V609, V614–V619, V624–V629, V634–V639, V644–V649, V654– V659, V664–V669, V674–V679, V684–V689, V694–V699, V704–V709, V714–V719, V724–V729, V734–V739, V744–V749, V754–V759, V764–V769, V774–V779, V784–V789, V794–V799, V803–V805, V811, V821, V830–V833, V840–V843, V850– V853, V860–V863, V870–V878, V892. †Rest of V = V-series MINUS §. | | | |

Reference List

1. Ezzati M, Lopez A, Rodgers A, Murray CJL: **Comparative quantification of health risks. Global and regional burden of disease attributable to selected major risk factors**. Geneva, Switzerland: World Health Organization; 2004.

2. Harris EC, Barraclough B: **Excess mortality of mental disorder**. *Br J Psychiatry* 1998, **173**:11-53.

3. Lönnroth K, Williams B, Stadlin S, Jaramillo E, Dye C: **Alcohol use as a risk factor for tuberculosis - a systematic review**. *BMC Public Health* 2008, **8**:289.

4. Rehm J, Mathers C, Popova S, Thavorncharoensap M, Teerawattananon Y, Patra J: **Global burden of disease and injury and economic cost attributable to alcohol use and alcohol use disorders**. *Lancet* 2009, **373**(9682):2223-2233.

5. Gmel G, Shield K, Rehm J: **Developing a methodology to derive alcohol-attributable fractions for HIV/AIDS mortality based on alcohol's impact on adherence to antiretroviral medication**. *Popul Health Metr* 2011, **9**(1):5.

6. Baan R, Straif K, Grosse Y, Secretan B, El Ghissassi F, Bouvard V, Alteri A, Cogliano V, On behalf of the W. H. O. International Agency for Research on Cancer monograph working group: **Carcinogenicity of alcoholic beverages**. *Lancet Oncology* 2007, **8**(4):292-293.

7. International Agency for Research on Cancer: *IARC Monograph 96 on the Evaluation of Carcinogenic Risks to Humans. Alcoholic beverage consumption and ethyl carbamate (urethane)*. Lyon, France: International Agency for Research on Cancer (IARC); 2010.

8. Corrao G, Bagnardi V, Zambon A, La Vecchia C: **A meta-analysis of alcohol consumption and the risk of 15 diseases**. *Prev Med* 2004, **38**:613-619.

9. Baliunas D, Taylor B, Irving H, Roerecke M, Patra J, Mohapatra S, Rehm J: **Alcohol as a risk factor for type 2 diabetes - A systematic review and meta-analysis**. *Diabetes Care* 2009, **32**(11):2123-2132.

10. Samokhvalov AV, Irving H, Mohapatra S, Rehm J: **Alcohol consumption, unprovoked seizures and epilepsy: a systematic review and meta-analysis**. *Epilepsia* 2010, **51**(7):1177-1184.

11. Taylor B, Irving HM, Baliunas D, Roerecke M, Patra J, Mohapatra S, Rehm J: **Alcohol and hypertension: gender differences in dose-response relationships determined through systematic review and meta-analysis**. *Addiction* 2009, **104**(12):1981-1990.

12. Roerecke M, Rehm J: **The cardioprotective association of average alcohol consumption and ischaemic heart disease: a systematic review and meta-analysis?** *Addiction* 2012, **107**(7):1246-1260.

13. Roerecke M, Rehm J: **Irregular heavy drinking occasions and risk of ischemic heart disease: a systematic review and meta-analysis**. *Am J Epidemiol* 2010, **171**(6):633-644.

14. Samokhvalov AV, Irving HM, Rehm J: **Alcohol as a risk factor for atrial fibrillation: a systematic review and meta-analysis**. *Eur J Cardiovasc Prev Rehabil* 2010, **17**(6):706-712.

15. Patra J, Taylor B, Irving H, Roerecke M, Baliunas D, Mohapatra S, Rehm J: **Alcohol consumption and the risk of morbidity and mortality from different stroke types - a systematic review and meta-analysis**. *BMC Public Health* 2010, **10**(1):258.

16. Rehm J, Taylor B, Mohapatra S, Irving H, Baliunas D, Patra J, Roerecke M: **Alcohol as a risk factor for liver cirrhosis - a systematic review and meta-analysis**. *Drug Alcohol Rev* 2010, **29**(4):437-445.

17. Irving HM, Samokhvalov A, Rehm J: **Alcohol as a risk factor for pancreatitis. A systematic review and meta-analysis**. *JOP* 2009, **10**(4):387-392.

18. Samokhvalov AV, Irving HM, Rehm J: **Alcohol consumption as a risk factor for pneumonia: systematic review and meta-analysis**. *Epidemiol Infect* 2010, **138**(12):1789-1795.
