# Supplementary material for: The potential impact of increased treatment rates for alcohol dependence in the United Kingdom in 2004
Source: BMC Health Serv Res. 2014 Feb 5;14:53. doi: 10.1186/1472-6963-14-53 (PMC3923387; doi:10.1186/1472-6963-14-53)
Supplement: Additional file 3 — Percentage of all alcohol-attributable deaths avoided by increasing AD treatment coverage to 20% (sensitivity analyses). [file 1472-6963-14-53-S3.docx]

Additional file 3. Percentage of all alcohol-attributable deaths avoided by increasing alcohol dependence treatment coverage to 20%


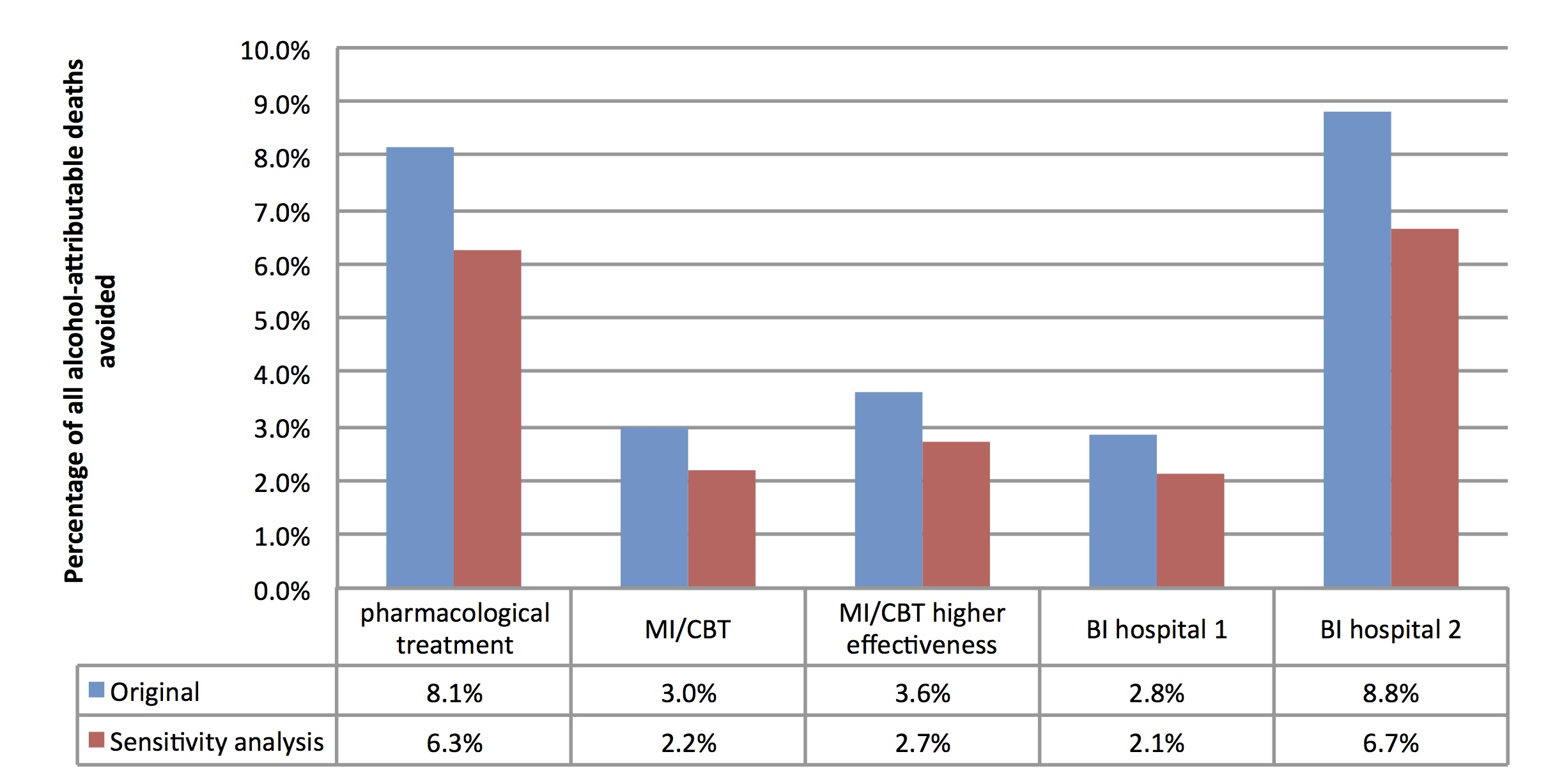


Figure A1. Percentage of all alcohol-attributable deaths avoided in the UK for men based on two different prevalence estimates for AD (assuming a 20% treatment coverage rate) for pharmacological treatment, Motivational Interviewing/Cognitive Behavioural (MI/CBT) (based on the lower (MBI/CT 1) and upper reported estimates (MBI/CT 2)), and Brief Interventions (BI) treatment (based on the resulting reduction in alcohol consumption (BI hospital 1) and the resulting reduction in mortality (BI hospital 2)).


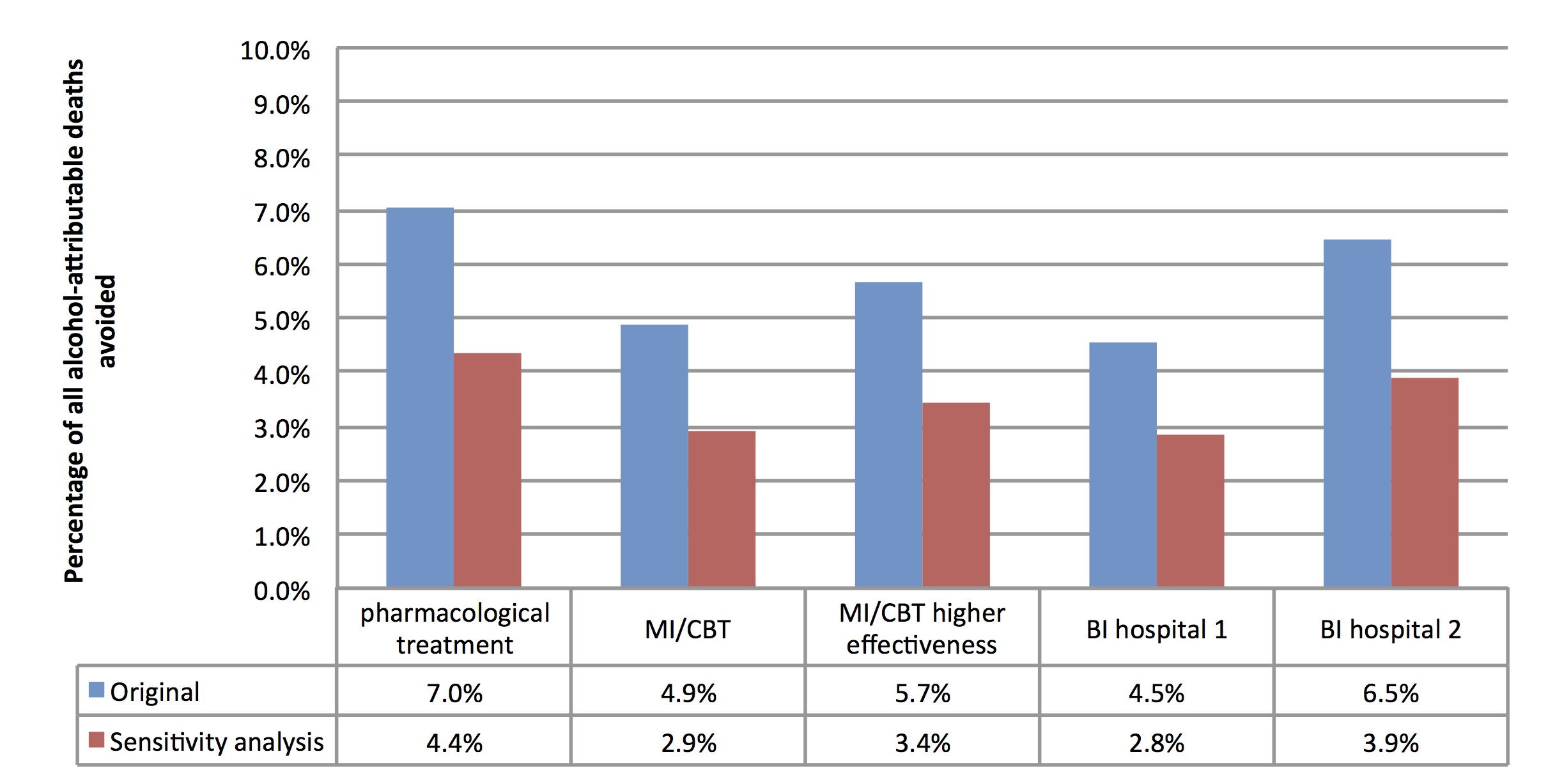


Figure A2. Percentage of all alcohol-attributable deaths avoided in the UK for women based on two different prevalence estimates for AD (assuming a 20% treatment coverage rate) for pharmacological treatment, Motivational Interviewing/Cognitive Behavioural (MI/CBT) (based on the lower (MBI/CT 1) and upper reported estimates (MBI/CT 2)), and Brief Interventions (BI) treatment (based on the resulting reduction in alcohol consumption (BI hospital 1) and the resulting reduction in mortality (BI hospital 2)).
